# Bilirubin Binding to PPARα Inhibits Lipid Accumulation

**DOI:** 10.1371/journal.pone.0153427

**Published:** 2016-04-12

**Authors:** David E. Stec, Kezia John, Christopher J. Trabbic, Amarjit Luniwal, Michael W. Hankins, Justin Baum, Terry D. Hinds

**Affiliations:** 1 Center for Hypertension and Personalized Medicine, Department of Physiology & Pharmacology, University of Toledo College of Medicine, Toledo, OH, 43614, United States of America; 2 Center for Drug Design and Development, University of Toledo College of Pharmacy and Pharmaceutical Sciences, Toledo, OH, 43614, United States of America; 3 Cardiovascular-Renal Research Center, Department of Physiology and Biophysics, University of Mississippi Medical Center, 2500 North State St, Jackson, Mississippi, 39216, United States of America; 4 North American Science Associates, Inc. (NAMSA), 6750 Wales Rd, Northwood, Ohio, 43619, United States of America; INRA, FRANCE

## Abstract

Numerous clinical and population studies have demonstrated that increased serum bilirubin levels protect against cardiovascular and metabolic diseases such as obesity and diabetes. Bilirubin is a potent antioxidant, and the beneficial actions of moderate increases in plasma bilirubin have been thought to be due to the antioxidant effects of this bile pigment. In the present study, we found that bilirubin has a new function as a ligand for PPARα. We show that bilirubin can bind directly to PPARα and increase transcriptional activity. When we compared biliverdin, the precursor to bilirubin, on PPARα transcriptional activation to known PPARα ligands, WY 14,643 and fenofibrate, it showed that fenofibrate and biliverdin have similar activation properties. Treatment of 3T3-L1 adipocytes with biliverdin suppressed lipid accumulation and upregulated PPARα target genes. We treated wild-type and PPARα KO mice on a high fat diet with fenofibrate or bilirubin for seven days and found that both signal through PPARα dependent mechanisms. Furthermore, the effect of bilirubin on lowering glucose and reducing body fat percentage was blunted in PPARα KO mice. These data demonstrate a new function for bilirubin as an agonist of PPARα, which mediates the protection from adiposity afforded by moderate increases in bilirubin.

## Introduction

Recent investigations have revealed that increased bilirubin levels are positively associated with a leaner phenotype and are protective of the vasculature system. However, the mechanism is unknown. Beyond functioning as an antioxidant [1], bilirubin has no known physiologic function. Water-insoluble, unconjugated bilirubin normally travels through the bloodstream to the liver, where it is converted into a water-soluble, conjugated form by the uridine diphosphate glucuronyltransferase (UGT) system and then excreted into bile [2]. Mutations in the UGT system result in elevated plasma levels of unconjugated bilirubin. Gilbert’s syndrome (GS) is the most common hereditary cause of hyperbilirubinemia, affecting approximately 5% to 10% of the population. GS is the result of reduced activity of the UGT enzyme, UGT1A1, resulting in higher plasma bilirubin levels. GS patients exhibiting mildly elevated levels of bilirubin were found to have a reduced risk of coronary artery disease (CAD) and a lower contingency for future heart disease [3]. Hypertensive patients with established CAD have significantly lower bilirubin levels [4, 5], which was also shown in diabetic patients with CAD [6]. Andersson et al. investigated short-term weight loss in obese high-risk cardiovascular patients and found that bilirubin increased as body weight decreased [7]. Bilirubin may be particularly effective in reducing adiposity since it readily enters the lipid environment [2, 8], which may serve to protect patients with the metabolic syndrome, as it was shown that higher bilirubin levels were paralleled with lower visceral obesity [9]. This correlated with the observation that obese patients with elevated insulin and visceral adiposity had decreased levels of bilirubin [10]. Interestingly, GS patients have improved adipocyte function and vascular protection [11–15]. The effects of bilirubin on adipocyte function have not been investigated. We have recently shown that increasing the production of bilirubin in obese mice resulted in the elevation of the fat burning nuclear receptor, PPARα, reducing body weight and blood glucose [16]. In this study, we show for the first time that bilirubin directly binds to activate PPARα, which increases target genes to reduce adiposity. The ability of bilirubin to act as an activator of nuclear hormone receptors such as PPARα is a novel function and may explain the beneficial effects of moderate increases in plasma bilirubin levels that have been observed in patients with GS.

## Methods

### Animals

The experimental procedures and protocols of this study conform to the National Institutes of Health Guide for the Care and Use of Laboratory Animals and were approved by the Institutional Animal Care and Use Committee of the University of Mississippi Medical Center in accordance with the *NIH Guide for the Care and Use of Laboratory Animals*. Studies were performed on 16-week-old male PPARα knockout and wild-type mice on a C57 genetic background purchased from Jackson Labs (Bar Harbor, ME). Mice were housed under standard conditions and allowed full access to a control 17% fat diet (Teklad 22/5 rodent diet, #860, Harland Laboratories, Inc., Indianapolis, IN) for 4 weeks. After this time, mice were switch to a 60% high fat diet (diet # D12492, Research Diets, Inc., New Brunswick, NJ) for an additional 6 weeks. All mice had free access to water. Animal activity and grooming were monitored daily to assess overall animal health. Animals were housed in a temperature-controlled environment with 12 h dark-light cycle. During treatment, mice were injected with either bilirubin (30 mg/kg, i.p.) or fenofibrate (90 mg/kg, i.p.) every 48 hours over the last week of the high fat diet. Control mice were not treated. Mice were euthanized on the last day of the study with overdose of isoflurane anesthesia in specially adapted cylinders followed by cervical dislocation and organ collection. Organs were also weighed at this time. Bilirubin was prepared in 0.1 M NaOH (pH 7.7) and fenofibrate was prepared in corn oil.

### Body Composition (EchoMRI)

Body composition changes were assessed at the end of the study using magnetic resonance imaging (EchoMRI-900TM, Echo Medical System, Houston, TX). MRI measurements were performed in conscious mice placed in a thin-walled plastic cylinder with a cylindrical plastic insert added to limit movement of the mice. Mice were briefly submitted to a low intensity electromagnetic field and fat mass, lean mass, free water, and total water were measured.

### Fasting Glucose and Insulin

Following an 8 hour fast, a blood sample was obtained via orbital sinus under isoflorane anesthesia. Blood glucose was measured using an Accu-Chek Advantage glucometer (Roche, Mannheim, Germany). Fasting plasma insulin concentrations were determined by ELISAs (Linco Insulin ELISA kit) as previously described [17].

#### Measurement of plasma bilirubin, alanine aminotransferase (ALT) and aspartate aminotransferase (AST)

Total bilirubin was measured from 20 μL of plasma using the Total Bilirubin IR700 Assay Kit (Synermed, Westfield, IN) according to the manufacturer instructions. The bilirubin assay was calibrated with a standard curve derived from a bilirubin solution provided by the manufacturer. Total bilirubin was determined by measurement at 700 nm on a plate reader. Plasma samples from individual mice were measured in duplicate and then averaged. The concentrations are expressed as mg/dL. Plasma alanine aminotransferase (ALT) and aspartate aminotransferase (AST) levels were determined in 50 μL of plasma by colorimetric assay (Cobas, Roche Diagnostics, Indianapolis, IN). Assays were performed according to manufactures guidelines and samples read on a Roach Cobas c501 analyzer. The concentrations are expressed as units/L.

### Measurement of Plasma FGF21

Plasma levels of FGF21 were measured from 50 μL of plasma using a specific mouse/rat FGF21 ELISA (Quantikine ELISA, R & D Systems, Minneapolis, MN) according to manufacture’s instructions. The FGF-21 ELISA was calibrated with a standard curve derived from a mouse/rat FGF21 standard provided by the manufacturer. FGF21 levels were measured in duplicate from individual mice and FGF21 levels determined by measurement at 450 nm on a plate reader. The concentrations are expressed as ng/mL.

### Cell Lines and Culture

The mouse 3T3-L1 preadipocyte, Hepa1c1c7, and Cos7 green kidney monkey cells were routinely cultured and maintained in Dulbecco’s Modified Eagles’s Medium (DMEM) containing 10% bovine calf serum or FBS with 1% pencillin-streptomycin. The vector and PPARα 3T3-L1 cell lines were grown as previously described [18].

### Promoter Reporter Assays

Expression vector for PPARα-pcDNA3.1+ was constructed as previously described [18]. A PPARα minimal promoter PPRE-3tk-luc activity was measured by luciferase, and pRL-CMV Renilla reporter for normalization to transfection efficiency. Transient transfection was achieved using GeneFect (Alkali Scientific, Inc.). Twenty-four-hour post-transfected cells were lysed, and the luciferase assay was performed using the Promega dual luciferase assay system (Promega, Madison, WI).

### In Silico Molecular Modeling and Docking Analysis of Bilirubin

Docking studies were carried out using Tripos’s Surflexdock suite on SYBYL-X molecular modeling package. Briefly, PPARα x-ray crystal structure was imported from RCSB Protein Data Bank (PDB ID: 2P54) [19]. The protein structure was prepared using SYBYL’s Biopolymer tool where terminal groups were appropriately functionalized, and the acidic residues were maintained at the physiological protonated state. The standard AMBER and MMFF94 charges were assigned to the bio-molecule and the small molecules, respectively. The docking model was internally validated where the ‘crystal structure bound ligand’ was first energy minimized using default setting followed by docking on the receptor site using the dock model. The top scoring conformation of ligand was aligned with the ‘bound crystal structure of the ligand.’ The two conformations-the docked model conformation and the crystal conformation- were aligned one-over-the-other. Similarly, bilirubin chemical structure was sketched and energy minimized prior to docking into the receptor site.

### EAH Sepharose^TM^ 4B Coupled to Either Bilirubin, Biliverdin or WY 14,643

The ligand coupling was performed according to the GE Healthcare instructions (71-7097-00 AE, pg. 6) for EAH Sepharose^TM^ 4B. The procedure in the online instructions was titled “A general ligand coupling procedure.” In summary, concentrations of ligands (bilirubin, biliverdin or WY 14,643) were 5 times the molar excess calculated for the free amine groups (12 μmol/ mL drained matrix). Resin coupling procedures were conducted in a DMF/ H_2_O solvent system (1:1) with a final concentration of 0.1 M EDC•HCl. Suspensions were rotated end-over-end for 24–36 h at room temperature (however, see note in next paragraph regarding bilirubin solubility). Upon completion, resins were washed according to the GE instructions (3 alternating washings with 0.5 M NaCl containing 0.1 M sodium acetate pH 4.5 and 0.5 M NaCl containing 0.1 M Tris pH 8) over a 10–15 μm fritted filter. As an additional step to the GE instructions, matrix-coupled bilirubin and biliverdin coupled preparations were further washed with 50% DMSO/H_2_O solutions (250 mL) to remove any unreacted ligand. The filtrate was nearly colorless after this step. For WY 14,643, 50% DMF/ H_2_O solutions (100 mL) were used to wash off any unreacted ligand. The resins were suspended in 20% EtOH/ H_2_O (15 mL) and stored at 4°C for 16 h in capped sample vials. The ligand-coupled resin settles overnight, and the supernatant was carefully decanted until minimal amounts of 20% EtOH/ H_2_O covered the resin. Aliquots (~ 1.5 mL) from each sample were suspended in the presence and absence of 1 M acetic acid, which is recommended to block unreacted free amines on the resin that did not react. Aliquots for samples designated “+AA” were subjected to 1 M acetic acid overnight, while “-AA” describes no acetic acid treatment and simply suspended in 20% EtOH/ H_2_O. In the case for +AA samples, after 16 h, the acetic acid solution was carefully decanted and then re-suspended in the storage solution (20% EtOH/ H_2_O). When comparing the results of resin preparations in the presence and absence of AA, we determined that the AA treatment had no effect on bilirubin binding PPARα. However, the AA treatment attenuated WY 14,643 binding PPARα. All samples were stored in 20% EtOH/ H_2_O before use.

Due to the limited solubility of bilirubin in most organic solvents, we compared preparations of resins in which bilirubin solutions were either heated (75°C for 90 min) or not heated in DMF/H_2_O prior to addition of EDC•HCl. This was to help increase the solubility of bilirubin in solution. It is noteworthy to point out that ethylene glycol, an ideal solvent suggested by GE, was not an appropriate co-solvent due to bilirubin’s limited solubility. The decrease in PPARα binding in resin preparations where bilirubin was heated suggests bilirubin is less stable with heating, which is known. Ultimately, a sufficient concentration of bilirubin was achieved at room temperature. Both biliverdin and WY 14,643 were readily soluble in organic solvents and application of heat was not attempted. Bilirubin was purchased commercially from Frontier Scientific. Biliverdin and WY 14,643 were purchased from Sigma-Aldrich.

#### Whole Cell Extraction

Cells were washed and collected in 1X PBS followed by centrifugation at 1500 X g for 10 min. The supernatant was discarded and the pellet was re-suspended in 1X PBS. After a short spin at 20,800 X g for 5 min at 4°C the pellet was rapidly frozen on dry ice ethanol mix and stored at -80°C for 30 min. The frozen pellet was then re-suspended in 3 volumes of cold whole cell extract buffer (20mM HEPES, 25% glycerol, 0.42M NaCl, 0.2mM EDTA, pH 7.4) with protease inhibitors and incubated on ice for 10 min. The samples were centrifuged at 100,000 X g for 5 min at 4°C. Protein levels were measured spectrophotometrically by a Nanodrop 2000 (Thermo fisher Scientific, Wilmington, DE). The supernatants were either stored at –80°C or used immediately for Western analysis to determine protein expression levels.

#### Quantitative Real-Time PCR Analysis

Total RNA was extracted from mouse tissues using 5-Prime PerfectPure RNA Cell Kit (Fisher Scientific Company, LLC). Total RNA was read on a NanoDrop 2000 spectrophotometer (Thermo Fisher Scientific, Wilmington, DE) and cDNA was synthesized using High Capacity cDNA Reverse Transcription Kit (Applied Biosystems). PCR amplification of the cDNA was performed by quantitative real-time PCR using TrueAmp SYBR Green qPCR SuperMix (Advance Bioscience). The thermocycling protocol consisted of 10 min at 95°C, 40 cycles of 15 sec at 95°C, 30 sec at 60°C, and 20 sec at 72°C and finished with a melting curve ranging from 60–95°C to allow distinction of specific products. Normalization was performed in separate reactions with primers to GAPDH.

### Generation of Lentiviral Constructs

To establish a 3T3-L1 or Hepa1c1c7 cell lines that have PPARα stably overexpressed, mouse PPARα cDNA was ligated into the NotI/BamHI sites of the pQXCIP vector and transformed in DH5α cells (Invitrogen, Carlsbad, CA). The construct was co-transfected together with vectors expressing gag-pol, REV, and VSV-G into 293FT cells (Invitrogen) to generate a third generation lentiviral construct. Transfection was achieved using GeneFect (Alkali Scientific, Inc.) using 100 ng total DNA per cm^2^ of the growth plate or well. The supernatants were harvested, and the cell debris was removed by centrifugation at 2000xg. The supernatant was used to infect 3T3-L1 or Hepa1c1c7 cells after addition of polybrene (5 ng/ml, Sigma Chemical Co., St. Louis, MO) to establish cell lines with stable overexpression of a PPARα overexpressing (3T3-PPARα) or expressing empty vector (3T3-Vector). After 72 h the cells were selected with puromycin, and positive cells were confirmed by Western blotting and used for experiments.

#### Adipogenesis Assay

Adipogenic differentiation of 3T3-L1 cells was achieved by treatment with 1 μM Dex, 830 nM insulin, and 100 μM isobutylmethylxanthine in 10% FBS until Day 9 [20–22]. Upon differentiation, cells were stained with Nile Red to visualize lipid content, and densitometry was used as a direct measure. Total RNA extracted from Nile Red stained cells was used for real time PCR analysis.

#### Gel Electrophoresis and Western Blotting

Whole cell extracts (WCE) were prepared by freezing the cell pellet overnight at −80°C. The pellet was then resuspended in 3 volumes of WCE buffer (20 mM HEPES, 0.42 M NaCl, 0.2 M EDTA, 25% glycerol, pH 7.4) plus protease inhibitor cocktail and incubated on ice for ten min followed by 100,000 × g centrifugation at 4°C. Protein samples were resolved by SDS polyacrylamide gel electrophoresis and electrophoretically transferred to Immobilon-FL membranes. Membranes were blocked at room temperature for 1 hour in TBS [TBS; 10 mM Tris-HCl (pH 7.4) and 150 mM NaCl] containing 3% BSA. Subsequently, the membrane was incubated overnight at 4°C with PPARα or HSP90 antibodies (Santa Cruz Biotechnology, Dallas, Texas) After three washes in TBST (TBS plus 0.1% Tween 20), the membrane was incubated with an infrared anti-rabbit (IRDye 800, green) or anti-mouse (IRDye 680, red) secondary antibody labeled with IRDye infrared dye (LI-COR Biosciences) (1:15,000 dilution in TBS) for 2 hours at 4°C. Immunoreactivity was visualized and quantified by infrared scanning in the Odyssey system (LI-COR Biosciences).

#### Statistical Analysis

Data were analyzed with Prism 6 (GraphPad Software, San Diego, CA) using analysis of variance combined with Tukey’s post-test to compare pairs of group means or unpaired *t* tests. Results are expressed as mean ± SEM. Additionally, one-way ANOVA with a least significant difference post hoc test was used to compare mean values between multiple groups, and a two-tailed, and a two-way ANOVA was utilized in multiple comparisons, followed by the Bonferroni post hoc analysis to identify interactions. *p* values of 0.05 or smaller were considered statistically significant.

## Results and Discussion

**Fig 1:**
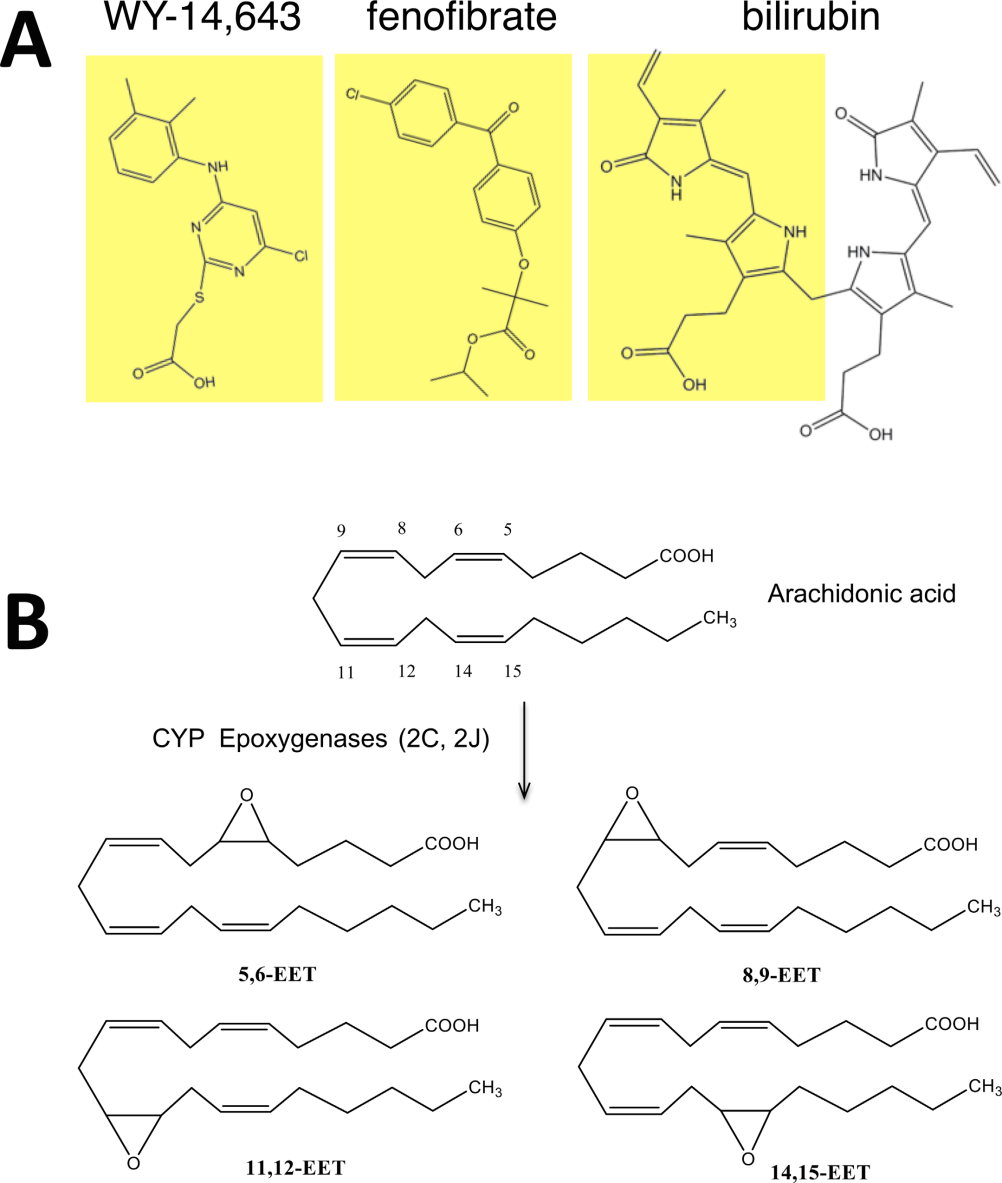
Structural of PPARα ligands. **(A)** Comparison of structures of WY 14, 643, fenofibrate and bilirubin. **(B)** Arachidonic acid is the precursor for CYP epoxygenase (2C and 2J) production of 5,6-, 8,9-, 11,12-, and 14, 15- epoxyeicosatrienoic acids (EETs).

**Fig 2:**
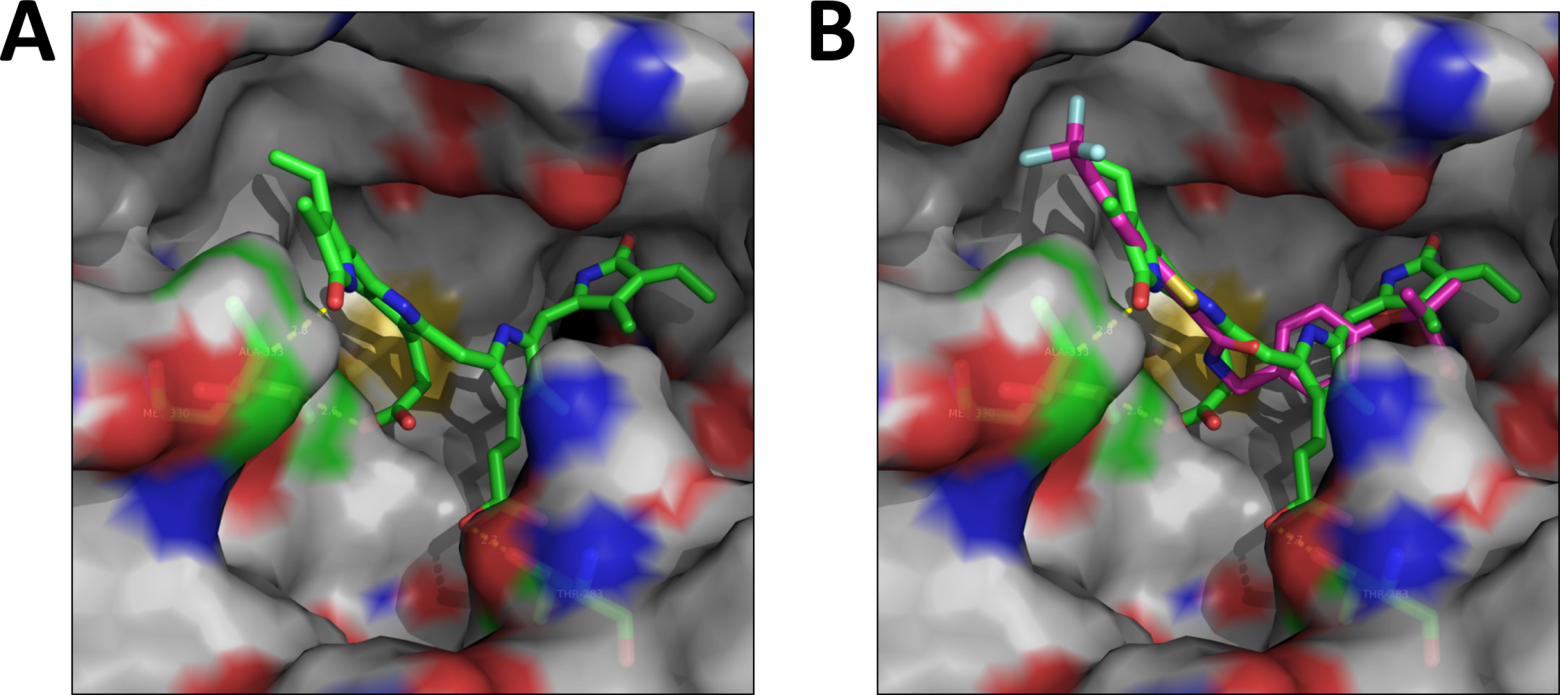
Bilirubin binds to the ligand-binding pocket of PPARα. **(A)** Bilirubin docked into PPARα binding pocket. **(B)** Bilirubin binds in the same site occupied by the known PPARα ligand GW735 [19]. Bilirubin and the ligand are depicted in green and magenta carbon skeleton, respectively.

Bilirubin plasma levels have been shown to be inversely correlated with lipid and glucose, and increasing levels have been shown to be beneficial for obesity, type II diabetes, and cardiovascular disease. We have recently shown that cobalt protoporphyrin (CoPP) treated mice had higher levels of bilirubin and increased PPARα expression [16]. Therefore, we wanted to deduce if bilirubin may directly bind to active the nuclear receptor. The aim of this study was to determine if bilirubin can directly bind to activate PPARα regulated gene activity, which could represent a novel pathway to explain the lipid lowering properties of bilirubin. A number of synthetic drugs have been developed as PPARα agonists, including WY 14,643 and fibrates that are used to treat hyperlipidemia. Upon comparison of WY 14,643 and fenofibrate, we realized that PPARα ligands have structural similarities to bilirubin (**Fig 1A**), potentially making bilirubin a ligand that could activate the low fidelity ligand-binding pocket of PPARα. There have been numerous endogenous ligands also identified for PPARα that includes several unsaturated fatty acids and their derivatives such as epoxyeicosatrienoic acids (EETs). PPARα has been shown to have anti-tumorigenic properties that are mediated by arachidonic acid epoxygenase [23]. The CYP2C and CYP2J epoxygenases metabolize arachidonic acid to 5,6-, 8,9-, 11,12-, and 14, 15-EETs (**Fig 1B**), which have been shown to bind and activate PPARα induced gene activity [24, 25]. However, the structures of the synthetic and endogenous PPARα ligands are diverse. An *in silico* modeling/docking analysis showed that bilirubin docks well into the ligand-binding pocket of PPARα (**Fig 2A**). Bilirubin binds to the same site occupied by the known PPARα ligand GW735 [19] (**Fig 2B**). A comparison of the two structures, the docked model conformation and the crystal structure of GW735, showed that they aligned one-over-the-other indicating tight binding in the docking model. Also, bilirubin exploits some additional interaction with receptor residues such as the H-bonding interaction between Threonine 223 and the carboxylate group of bilirubin indicating a stronger binding. Furthermore, bilirubin engages with the receptor through a thermodynamically more stable ‘twist’ conformation. The receptor sites seem to have two relatively distinct binding pockets, a more lipophilic left zone and a more hydrophilic region on the right, which may cause the ligands to 'arch' and engage with the two sites.

**Fig 3:**
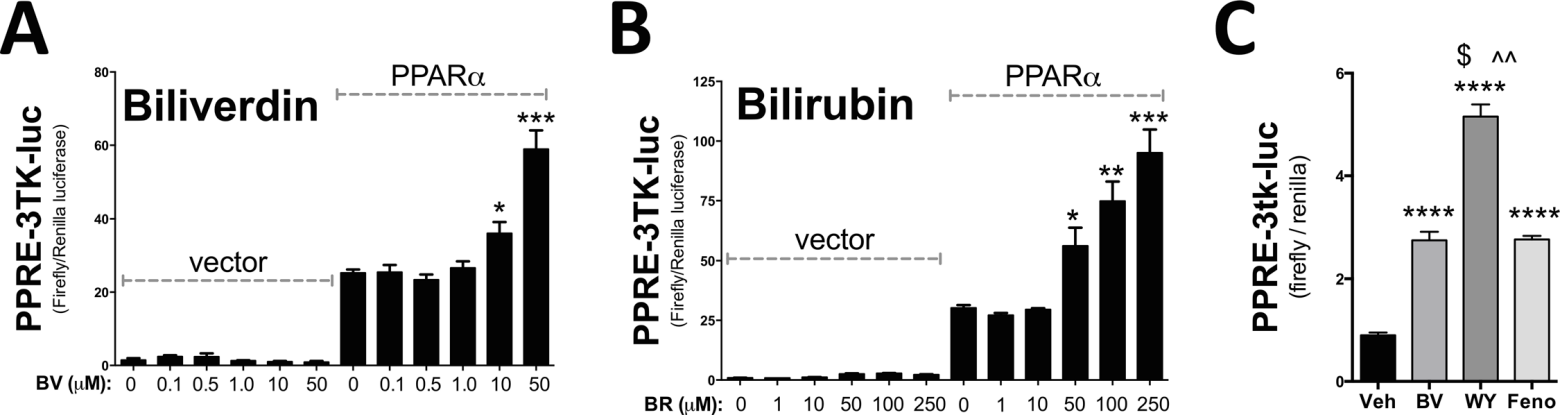
Bilirubin and biliverdin activate PPARα activity. To determine if bilirubin or biliverdin activate PPARα activity we used Cos7 cells that were transiently transfected with a minimal PPARα responsive promoter luciferase construct (PPRE-3tk-luc) for 24 hours along with empty vector and vector containing PPARα cDNA (overexpression). We treated for 24 hours with a dose dependent increase of biliverdin (BV) **(A)** or bilirubin (BR) **(B)**. *, *p* < 0.05; **, *p* < 0.01; ***, *p* < 0.001 (*versus* 0 μM PPARα); (±S.E.; *n* = 4). **(C)** To compare biliverdin (BV), WY 14,643 (WY), and fenofibrate (Feno) on PPARα activity, we use the minimal promoter PPRE-3tk-luc luciferase construct and treated for 24 hours with PPARα overexpressed and then treated with 50 μM each for 24 hours. ****, *p* < 0.0001 (*versus* 0 μM Veh); $ and ^^, *p* < 0.001 (*versus* 0 μM BV and Feno, respectively); (±S.E.; *n* = 4).

To determine if bilirubin or its precursor, biliverdin, can activate PPARα, a dose dependence of each molecule was performed in the presence and absence of PPARα (**Fig 3A & 3B**). In the absence of PPARα, biliverdin or bilirubin did not activate the PPRE-3tk-luc promoter. A dose dependence treatment showed that biliverdin and bilirubin significantly (p<0.05) increased PPARα activity. To compare biliverdin/bilirubin to known PPARα agonists, WY 14,643 and fenofibrate, we used the minimal PPRE-3tk-luc promoter to determine the level of activation among the ligands. Biliverdin, WY 14,643, and fenofibrate all significantly (p<0.0001) increased PPARα activity at the minimal luciferase promoter (**Fig 3C**). WY 14,643 significantly (p<0.001) increased promoter activity of PPRE-3tk-luc higher than biliverdin or fenofibrate. Interestingly, biliverdin and fenofibrate had the same level of PPARα activation.

**Fig 4:**
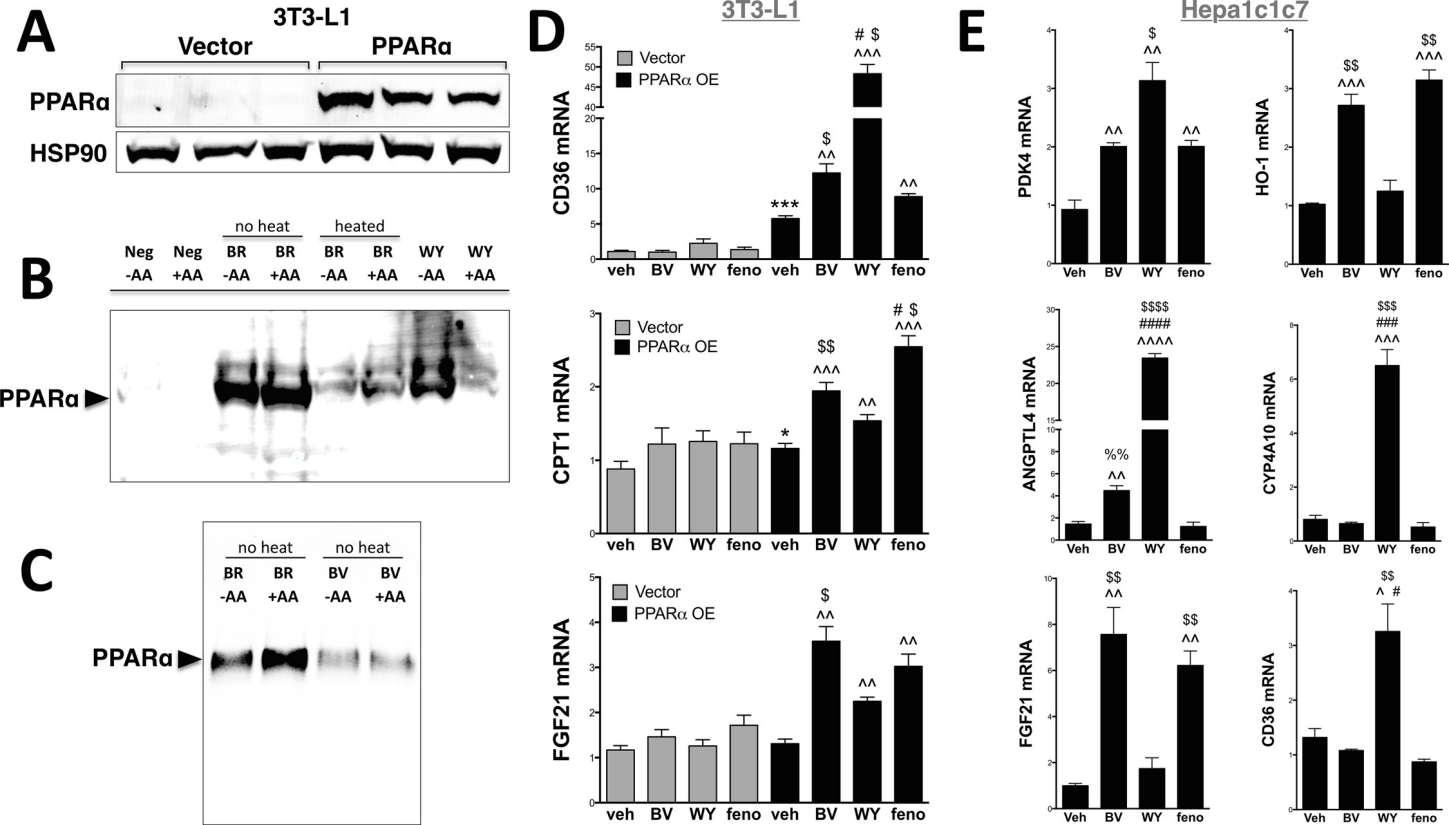
Bilirubin binds directly to PPARα to increase endogenous gene activity. **(A)** Western of PPARα and HSP90 in lentiviral overexpression of PPARα and vector in 3T3-L1 cells. **(B)** Bilirubin or WY 14,643 linked sepharose resins were used to determine direct binding to PPARα. **(C)** Bilirubin or biliverdin linked sepharose resins were used to determine direct binding to PPARα. **(D)** The PPARα overexpression and vector 3T3-L1 cells were treated for 24 hours with biliverdin (BV) (50 μM), WY 14,643 (WY) (50 μM), or fenofibrate (Feno) (50 μM). RNA was extracted and CD36, CPT1, and FGF21 expression was measured by Real-time PCR. ***, *p* < 0.001 (*versus* veh 3T3-Vector); ^, *p* < 0.05 (*versus* veh 3T3-PPARα); ^^, *p* < 0.01 (*versus* veh 3T3-PPARα); ^^^, *p* < 0.001 (*versus* veh 3T3-PPARα); $, *p* < 0.05 (*versus* WY 3T3-PPARα); $ $, *p* < 0.01 (*versus* WY 3T3-PPARα); #, *p* < 0.05 (*versus* BV 3T3-PPARα); (±S.E.; *n* = 3). **(E)** The mouse hepa1c1c7 liver cells overexpressing PPARα were treated in dialyzed FBS for 24 hours with biliverdin (BV) (50 μM), WY 14,643 (WY) (50 μM), or fenofibrate (Feno) (50 μM). RNA was extracted and mRNA expression was measured by Real-time PCR. ^, *p* < 0.05, ^^, *p* < 0.01, and ^^^, *p* < 0.001 (*versus* veh 3T3-PPARα); $, *p* < 0.05, $ $, *p* < 0.01, $ $ $, *p* < 0.001, $ $ $ $, *p* < 0.0001 (*versus* WY 3T3-PPARα); ###, *p* < 0.001, #, *p* < 0.01, ####, *p* < 0.0001 (*versus* BV 3T3-PPARα); (±S.E.; *n* = 3).

To show that bilirubin/biliverdin can bind PPARα as well as regulate endogenous genes, we constructed a stable cell line via lentivirus with PPARα cDNA overexpressed (PPARα OE) or vector control in 3T3-L1 cells (**Fig 4A**), as they have been shown to have low to no PPARα expression in the undifferentiated state [26]. First, to determine if bilirubin is directly binding to activate PPARα, we coupled the carboxylic acid group of either WY 14,643 or bilirubin to amino-functionalized sepharose beads (described in detail in the Methods). We used PPARα OE 3T3-L1 cells to perform pull-down assays to determine that PPARα directly binds bilirubin and WY 14,643 (**Fig 4B**). The pull-down results show that PPARα can directly bind to bilirubin and the known PPARα agonist, WY 14,643. To compare the binding of biliverdin and bilirubin to PPARα, we used PPARα OE 3T3-L1 cells for a pull-down assay with sepharose beads cross-linked with either bilirubin or biliverdin. Interestingly, bilirubin had preferential binding to PPARα compared to biliverdin (**Fig 4C**). The double bond linking the two dipyrrin-1-one functionalities of biliverdin may cause a rigidity not seen in bilirubin (where the two dipyrrin-1-one groups are linked by a saturated methylene group) and may not allow the bending/twisting in the conformation seen in Fig 2A. The thermodynamic stability of bilirubin as compared to structurally fixed biliverdin in the PPARα binding pocket may explain the difference in binding. Ultimately, these results suggest that biliverdin must be reduced to bilirubin intracellulary through the enzyme, biliverdin reductase (BVR) [2], to effect PPARα activity.

To determine endogenous PPARα gene regulatory activity, we treated vector controls (no PPARα) and PPARα OE 3T3-L1 cells with 50 μM biliverdin, fenofibrate, and WY 14,643 for 24 hours in dialyzed fatty acid-free media. Experiments were conducted with biliverdin because it has greater water solubility than bilirubin, and once inside the cell, it gets rapidly converted to bilirubin via the ubiquitous enzyme biliverdin reductase [2]. In **Fig 4D**, we show that WY 14,643 strongly induced expression of the anti-diabetic gene, Cluster of Differentiation 36 (CD36). Interestingly, biliverdin significantly (p<0.05) increased CD36 mRNA expression more than fenofibrate. PPARα has been shown to increase two major fatty acid oxidation genes, carnitine palmitoyltransferase 1 (CPT1) that is a mitochondrial enzyme that assists in the catalysis of long-chain fatty acids [16, 27] and the fibroblast growth factor 21 (FGF21) which is a hormone that sensitizes to glucose and reduces adiposity [16, 28–30]. Biliverdin and fenofibrate increased CPT1 and FGF21 expression more than WY 14,643 treatment, and biliverdin significantly (p<0.05) enhanced FGF21 mRNA higher than fenofibrate. To measure known PPARα controlled genes and the response from the different ligands in liver cells, we treated hepa1c1c7 mouse hepatocytes overexpressing PPARα with 50 μM biliverdin, fenofibrate, and WY 14,643 for 24 hours in dialyzed fatty acid-free media (**Fig 4E**). The FGF21 and heme oxygenase-1 (HO-1) mRNA response were both increased by biliverdin and fenofibrate but not with WY 14,643. Pyruvate dehydrogenase kinase 4 (PDK4) expression was increased with all the ligands but significantly (p<0.05) higher with WY 14,643 compared to biliverdin or fenofibrate. Another known PPARα regulated gene, angiopoietin-like 4 (ANGPTL4), which is involved in the release of fat from adipose [31], was significantly increased by biliverdin (p = 0.0035) and WY 14,643 (p<0.0001), but no response to fenofibrate was observed. Interestingly, CD36 and CYP4A10 mRNA in mouse liver cells only responded to WY 14,643 and not to biliverdin or fenofibrate.

These data suggest that there are variances in PPARα responses with these ligands and that biliverdin/bilirubin may have both anti-diabetic and antilipemic properties. The interaction of ligands with PPARα may be at different binding affinities, which can result in a slight conformational change that can potentially lead to higher regulatory activity of specific genes, possibly by cofactors that bind PPARα at promoters. These small variances can lead to divergent PPARα gene regulation, which has been shown with fenofibrate and WY 14,643 [32, 33]. We show in Figs 3C, 4D and 4E that biliverdin, WY 14,643, and fenofibrate activated PPARα at different levels, which may be due to different ligand binding affinity. Interestingly, the fibrates have been shown to be better at reducing inflammation than WY 14,643 and are typically used in treating inflammatory hyperlipidemia and fatty liver disease [26, 27, 34]. While WY 14,643 does reduce hyperlipidemia, it does not reduce hepatic inflammation [35]. However, WY 14,643 has been shown to be better at reducing blood glucose levels [36]. Because of these differences, PPARα ligands are considered to be either mostly anti-lipidemic or anti-diabetic. Bilirubin may have a similar effect when bound to PPARα and regulate specific gene activity that is both glucose-lowering and antilipemic.

**Fig 5:**
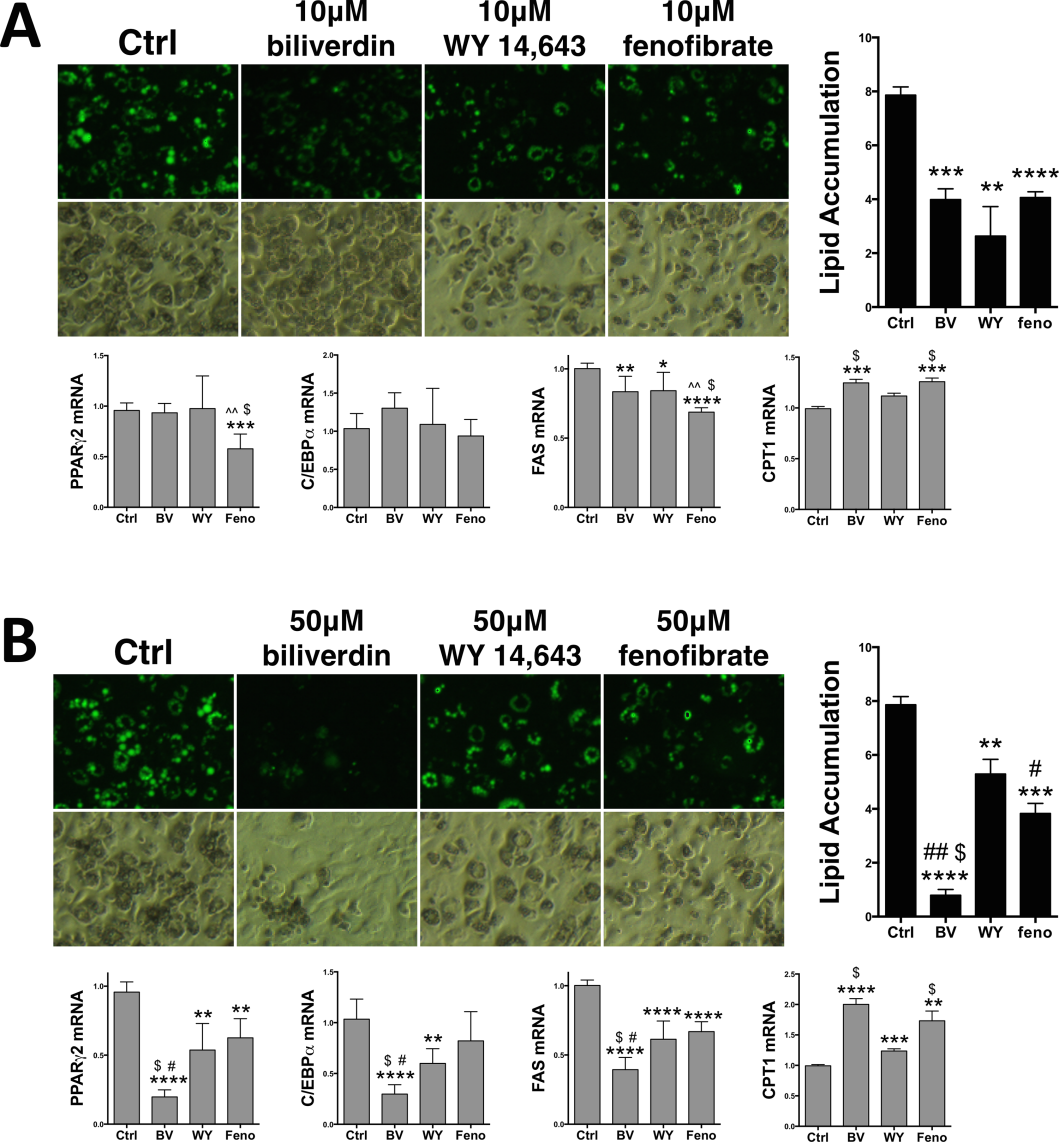
Biliverdin reduces lipid accumulation more than other PPARα ligands. **(A)** Lipid accumulation was measured by nile red staining (green) and densitometry in 3T3-L1 cells that were differentiated into mature adipocytes treated with vehicle (Ctrl), biliverdin (10 μM), WY 14,643 (10 μM), or fenofibrate (10 μM) over the 9 day protocol and Real-time PCR analysis of PPARγ2, C/EBPα, FAS, and CPT1. *, *p* < 0.05; **, *p* < 0.01; ***, *p* < 0.001; ****, *p* < 0.0001 (*versus* Ctrl); ^^, *p* < 0.05 (*versus* 10 μM WY); $, *p* < 0.05 (*versus* 10 μM feno) (±S.E.; *n* = 3). **(B)** Lipid accumulation was measured in 3T3-L1 cells that were differentiated into mature adipocytes treated with vehicle (Ctrl), biliverdin (50 μM), WY 14,643 (50 μM), or fenofibrate (50 μM) over the 9 day protocol and Real-time PCR analysis of PPARγ2, C/EBPα, FAS, and CPT1. (*versus* Ctrl) **, *p* < 0.01; ***, *p* < 0.001; ****, *p* < 0.0001; (*versus* 50 μM WY) #, *p* < 0.05; ##, *p* < 0.001; (*versus* 50 μM feno) $, *p* < 0.001 (±S.E.; *n* = 3).

To compare the antilipemic properties of the PPARα ligands, we used the 3T3-L1 cell model of adipogenesis and determined their effect on lipid accumulation. Treatment of biliverdin (10 μM), WY 14,643 (10 μM), and fenofibrate (10 μM) significantly (p<0.001) decreased lipid accumulation during adipogenesis (**Fig 5A**). Biliverdin reduced lipid accumulation by 49%, WY 14,643 by 56%, and fenofibrate by 48%. There was no significant difference among the PPARα ligands. PPARα has been previously shown to decrease adiposity by activation of the β-oxidation regulatory gene, CPT1 [16, 26]. Whereas, rapid loss of fat by leptin increases PPARα expression and fatty acid oxidation genes and decreases the de novo lipid producing enzyme, fatty acid synthase (FAS) [37, 38]. We show in Fig 5A, that biliverdin (10 μM), WY 14,643 (10 μM), and fenofibrate (10 μM) significantly (p<0.001) decreased expression of FAS. At this concentration, only fenofibrate inhibited expression of PPARγ2. However, both biliverdin and fenofibrate significantly (p<0.001) increased expression of CPT1. Treatment with the higher concentration of biliverdin (50 μM), WY 14,643 (50 μM), and fenofibrate (50 μM) significantly (p<0.001) decreased lipid accumulation during adipogenesis (**Fig 5B**). At the higher doses, biliverdin reduced lipid accumulation by 91%, WY 14,643 by 33%, and fenofibrate by 51%. Interestingly, biliverdin (50 μM) significantly (P<0.001) decreased more lipids compared to the same concentration of WY 14,643 and fenofibrate. These results indicate that biliverdin/bilirubin treatment in obese patients may have a stronger anti-lipogenic effect. It is also important to note that patients with Gilbert’s syndrome typically have about a 50% increased level of bilirubin in their plasma, which equates to 50 μM bilirubin [39]. Several large population studies have shown that individuals with serum bilirubin in the upper range of normal to slightly (50–100%) elevated levels are protected against hepatic steatosis, development of diabetes and the metabolic syndrome [9, 40–43]. In these studies, we show that 50 μM bilirubin substantially decreased lipid accumulation in the 3T3-L1 cells and enhanced PPARα activity at the minimal promoter and endogenous genes.

**Fig 6:**
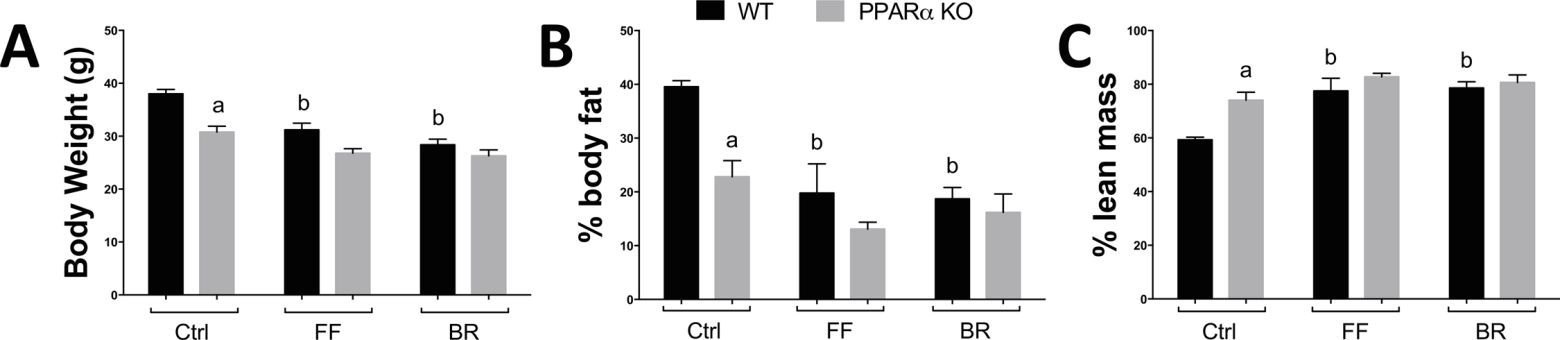
Bilirubin reduces body weight and body fat percentage. WT and PPARα KO mice were on a high fat diet for 6 weeks and treated with fenofibrate (FF) or bilirubin (BR) for seven days and body weight **(A)**, percent body fat **(B)**, and lean mass **(C)** were measured. a, *p* < 0.05 (KO *versus* WT Ctrl); b, *p* < 0.05 (WT FF or BR treated *versus* WT Ctrl) (±S.E.; *n* = 5).

**Fig 7:**
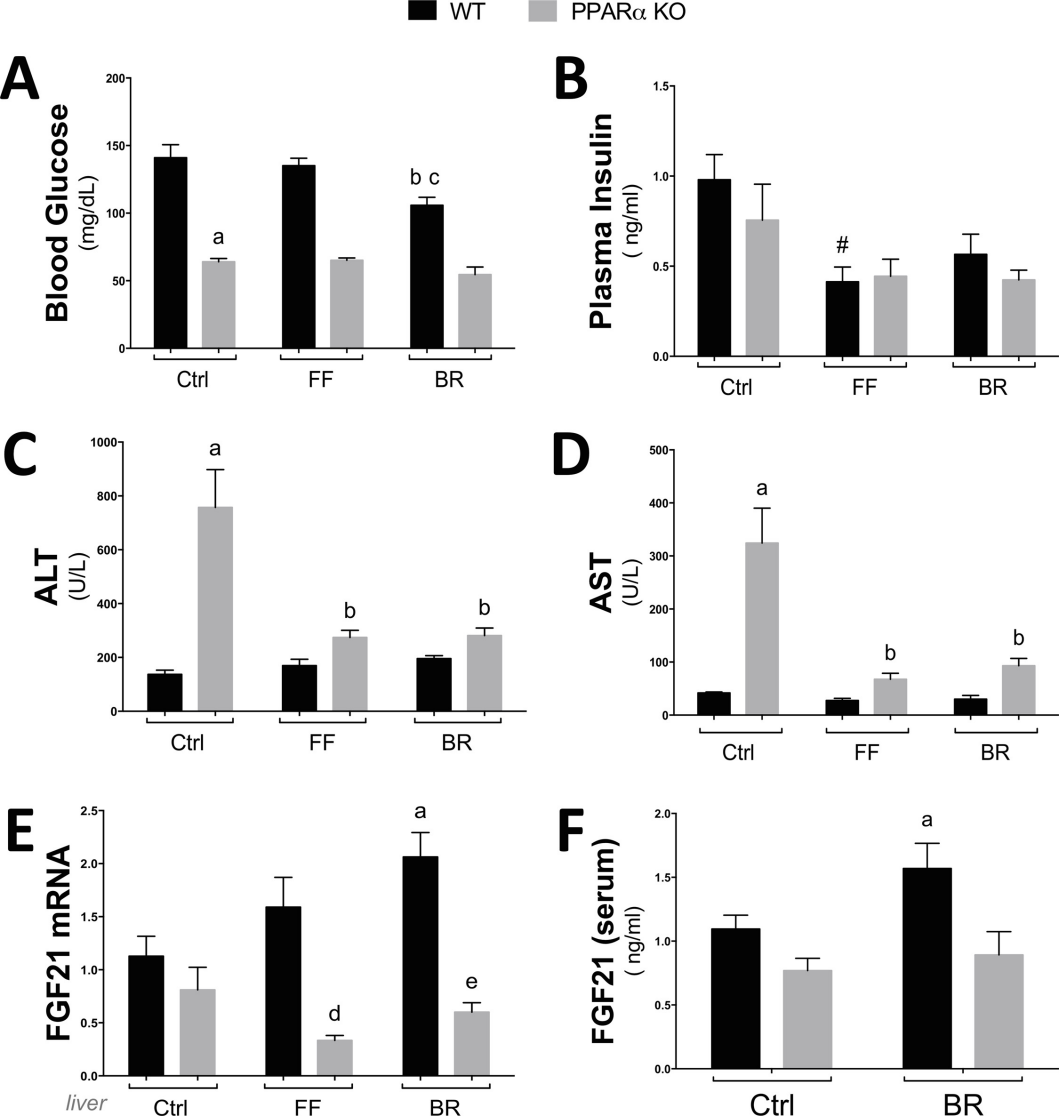
The glucose lowering affect of bilirubin is blunted in PPARα KO mice. WT and PPARα KO mice were on a high fat diet for 6 weeks and treated with fenofibrate (FF) or bilirubin (BR) for seven days and blood glucose **(A)**, plasma insulin **(B)**, alanine aminotransferase (ALT) **(C)**, aspartate aminotransferase (AST) **(D)**, and fibroblast growth factor (FGF21) mRNA in liver **(E)** and serum levels **(F)** were measured. a, *p* < 0.05 (KO *versus* WT Ctrl); b, *p* < 0.05 (WT FF or BR treated *versus* WT Ctrl); c, *p* < 0.05 (WT BR treated *versus* WT FF treated); d, *p* < 0.05 (KO FF treated *versus* WT FF); e, *p* < 0.05 (KO BR treated *versus* WT BR) (±S.E.; *n* = 5).

These results support that activation of PPARα in adipocytes increased fatty acid oxidation genes and decreased in de novo lipogenic enzymes. These processes are important in the management of obesity, which has been shown to be reduced with increased bilirubin levels in patients [10] and rodents [16]. Exercise induces fat utilization and burning by enhancing the β-oxidation pathway [44, 45]. Plasma bilirubin levels have been shown to increase with exercise [46], which may be to induce the burning of fat through PPARα induced β-oxidation. In **Fig 6A**, we show that mice treated with bilirubin (30 mg/kg) and fenofibrate (90 mg/kg) had significantly less body weight. However, bilirubin and fenofibrate had no effect on body weight in PPARα KO mice. The percentage body fat was decreased with fenofibrate and bilirubin, and lean mass was increased, which were not observed in PPARα KO mice (**Fig 6B & 6C**). Interestingly, bilirubin, but not fenofibrate, reduced blood glucose in the wild-type (WT) mice, and this effect was absent in the PPARα KO mice (**Fig 7A**). The plasma insulin levels were reduced with fenofibrate treatment but not significantly reduced with bilirubin (**Fig 7B**). Very high bilirubin levels have been shown in liver damage and failure. However, recent reports in Gilbert’s patients, with slightly elevated bilirubin levels, have shown that bilirubin has lipid-lowering and anti-diabetic protective properties. To determine if fenofibrate or bilirubin treatment altered the function of the liver of WT or PPARα KO mice, we measured alanine aminotransferase (ALT) and aspartate aminotransferase (AST) (**Fig 7C & 7D**), which are liver enzymes that are released into the bloodstream when it is damaged or diseased. ALT and AST were higher in the control PPARα KO mice, and significantly (p<0.05) decreased with bilirubin or fenofibrate treatments. Interestingly, WT mice had no change in AST or ALT with fenofibrate or bilirubin treatments. The ALT and AST levels may have been reduced in the PPARα KO mice by the antioxidant properties of bilirubin, but fenofibrate is not thought to have this property. The glucose lowering effect of bilirubin may be due to the PPARα activation of the FGF21 hormone, which is known to reduce blood glucose and adiposity [16, 28, 47–50]. Bilirubin significantly (p = 0.05) enhanced FGF21 mRNA levels in liver (**Fig 7E**) and serum (**Fig 7F**) but not in PPARα KO mice.

In conclusion, we have discovered that bilirubin can bind to enhance PPARα activity, which leads to the increase of lipid burning genes CPT1 and FGF21. Most studies have only considered bilirubin as an inert antioxidant that does not function to bind transcription factors as a ligand. Our studies clearly identify a novel role for bilirubin as an activator of the nuclear receptor family. These studies open new drug development concepts in the targeting of adiposity and the area of novel PPARα ligands. The main aspects studied for bilirubin have been on the inhibition of reactive oxygen species with little consideration given to it as a potential signaling molecule. Increased bilirubin levels in humans have already been correlated with reduced adiposity. Our studies clearly show that bilirubin has a regulatory role in the mediation of lipid metabolism through PPARα dependent signaling. These properties have not been previously known for bilirubin, especially the direct effect on PPARα gene regulation. Given that PPARα regulates genes involved in β-oxidation, increasing bilirubin levels by inhibiting UGT1A1 or by direct treatment may have a paramount role in the prevention of obesity. Thus, the bilirubin/PPARα axis is emerging as a major signaling paradigm regulating adiposity, which may also attenuate diabetes. Therapeutics inhibiting UGT1A1 may increase plasma bilirubin levels, as well as increase PPARα expression allowing for the management and prevention of obesity.

## References

[1] Stocker R, Yamamoto Y, McDonagh AF, Glazer AN, Ames BN. Bilirubin is an antioxidant of possible physiological importance. Science. 1987;235(4792):1043–6. . 3029864 10.1126/science.3029864

[2] O'Brien L, Hosick PA, John K, Stec DE, Hinds TD Jr. Biliverdin reductase isozymes in metabolism. Trends in endocrinology and metabolism: TEM. 2015;26(4):212–20. 10.1016/j.tem.2015.02.001 25726384 PMC4380527

[3] Vitek L, Jirsa M, Brodanova M, Kalab M, Marecek Z, Danzig V, et al Gilbert syndrome and ischemic heart disease: a protective effect of elevated bilirubin levels. Atherosclerosis. 2002;160(2):449–56. . 11849670 10.1016/s0021-9150(01)00601-3

[4] Ghem C, Sarmento-Leite RE, de Quadros AS, Rossetto S, Gottschall CA. Serum bilirubin concentration in patients with an established coronary artery disease. International heart journal. 2010;51(2):86–91. . 20379040 10.1536/ihj.51.86

[5] Yang XF, Chen YZ, Su JL, Wang FY, Wang LX. Relationship between serum bilirubin and carotid atherosclerosis in hypertensive patients. Internal medicine. 2009;48(18):1595–9. . 19755760 10.2169/internalmedicine.48.2286

[6] Chen YH, Chau LY, Chen JW, Lin SJ. Serum bilirubin and ferritin levels link heme oxygenase-1 gene promoter polymorphism and susceptibility to coronary artery disease in diabetic patients. Diabetes care. 2008;31(8):1615–20. 10.2337/dc07-2126 18443197 PMC2494663

[7] Andersson C, Weeke P, Fosbol EL, Brendorp B, Kober L, Coutinho W, et al Acute effect of weight loss on levels of total bilirubin in obese, cardiovascular high-risk patients: an analysis from the lead-in period of the Sibutramine Cardiovascular Outcome trial. Metabolism: clinical and experimental. 2009;58(8):1109–15. 10.1016/j.metabol.2009.04.003 . 19454355

[8] Zucker SD, Goessling W, Hoppin AG. Unconjugated bilirubin exhibits spontaneous diffusion through model lipid bilayers and native hepatocyte membranes. The Journal of biological chemistry. 1999;274(16):10852–62. . 10196162 10.1074/jbc.274.16.10852

[9] Choi SH, Yun KE, Choi HJ. Relationships between serum total bilirubin levels and metabolic syndrome in Korean adults. Nutrition, metabolism, and cardiovascular diseases: NMCD. 2011 10.1016/j.numecd.2011.03.001 . 21703835

[10] Torgerson JS, Lindroos AK, Sjostrom CD, Olsson R, Lissner L, Sjostrom L. Are elevated aminotransferases and decreased bilirubin additional characteristics of the metabolic syndrome? Obesity research. 1997;5(2):105–14. . 9112245 10.1002/j.1550-8528.1997.tb00650.x

[11] Cure E, Cicek Y, Cumhur Cure M, Yuce S, Kirbas A, Yilmaz A. The evaluation of relationship between adiponectin levels and epicardial adipose tissue thickness with low cardiac risk in Gilbert`s syndrome: an observational study. Anadolu kardiyoloji dergisi: AKD = the Anatolian journal of cardiology. 2013;13(8):791–6. 10.5152/akd.2013.266 . 24172837

[12] Kim DH, Burgess AP, Li M, Tsenovoy PL, Addabbo F, McClung JA, et al Heme oxygenase-mediated increases in adiponectin decrease fat content and inflammatory cytokines tumor necrosis factor-alpha and interleukin-6 in Zucker rats and reduce adipogenesis in human mesenchymal stem cells. The Journal of pharmacology and experimental therapeutics. 2008;325(3):833–40. 10.1124/jpet.107.135285 . 18334666

[13] Li M, Kim DH, Tsenovoy PL, Peterson SJ, Rezzani R, Rodella LF, et al Treatment of obese diabetic mice with a heme oxygenase inducer reduces visceral and subcutaneous adiposity, increases adiponectin levels, and improves insulin sensitivity and glucose tolerance. Diabetes. 2008;57(6):1526–35. 10.2337/db07-1764 . 18375438

[14] Kobashi C, Urakaze M, Kishida M, Kibayashi E, Kobayashi H, Kihara S, et al Adiponectin inhibits endothelial synthesis of interleukin-8. Circulation research. 2005;97(12):1245–52. 10.1161/01.RES.0000194328.57164.36 . 16269654

[15] Hopkins TA, Ouchi N, Shibata R, Walsh K. Adiponectin actions in the cardiovascular system. Cardiovascular research. 2007;74(1):11–8. 10.1016/j.cardiores.2006.10.009 17140553 PMC1858678

[16] Hinds TD Jr., Sodhi K, Meadows C, Fedorova L, Puri N, Kim DH, et al Increased HO-1 levels ameliorate fatty liver development through a reduction of heme and recruitment of FGF21. Obesity. 2013 10.1002/oby.20559 . 23839791 PMC3830593

[17] Hosick PA, AlAmodi AA, Storm MV, Gousset MU, Pruett BE, Gray W 3rd, et al Chronic carbon monoxide treatment attenuates development of obesity and remodels adipocytes in mice fed a high-fat diet. International journal of obesity. 2014;38(1):132–9. 10.1038/ijo.2013.61 23689359 PMC3760985

[18] Hinds TD Jr., Ramakrishnan S, Cash HA, Stechschulte LA, Heinrich G, Najjar SM, et al Discovery of glucocorticoid receptor-beta in mice with a role in metabolism. Molecular endocrinology. 2010;24(9):1715–27. 10.1210/me.2009-0411 20660300 PMC2940475

[19] Sierra ML, Beneton V, Boullay AB, Boyer T, Brewster AG, Donche F, et al Substituted 2-[(4-aminomethyl)phenoxy]-2-methylpropionic acid PPARalpha agonists. 1. Discovery of a novel series of potent HDLc raising agents. J Med Chem. 2007;50(4):685–95. 17243659 10.1021/jm058056x

[20] Hinds TD Jr., Stechschulte LA, Cash HA, Whisler D, Banerjee A, Yong W, et al Protein phosphatase 5 mediates lipid metabolism through reciprocal control of glucocorticoid receptor and peroxisome proliferator-activated receptor-gamma (PPARgamma). The Journal of biological chemistry. 2011;286(50):42911–22. 10.1074/jbc.M111.311662 21994940 PMC3234872

[21] Stechschulte LA, Hinds TD Jr., Ghanem SS, Shou W, Najjar SM, Sanchez ER. FKBP51 Reciprocally Regulates GRalpha and PPARgamma Activation via the Akt-p38 Pathway. Molecular endocrinology. 2014 10.1210/me.2014-1023 . 24933248 PMC4116593

[22] Stechschulte LA, Hinds TD Jr., Khuder SS, Shou W, Najjar SM, Sanchez ER. FKBP51 Controls Cellular Adipogenesis Through p38 Kinase-mediated Phosphorylation of GRalpha and PPARgamma. Molecular endocrinology. 2014 10.1210/me.2014-1022 . 24933247 PMC4116587

[23] Pozzi A, Popescu V, Yang S, Mei S, Shi M, Puolitaival SM, et al The anti-tumorigenic properties of peroxisomal proliferator-activated receptor alpha are arachidonic acid epoxygenase-mediated. The Journal of biological chemistry. 2010;285(17):12840–50. 10.1074/jbc.M109.081554 20178979 PMC2857132

[24] Wray J, Bishop-Bailey D. Epoxygenases and peroxisome proliferator-activated receptors in mammalian vascular biology. Experimental physiology. 2008;93(1):148–54. 10.1113/expphysiol.2007.038612 . 17872966

[25] Wray JA, Sugden MC, Zeldin DC, Greenwood GK, Samsuddin S, Miller-Degraff L, et al The epoxygenases CYP2J2 activates the nuclear receptor PPARalpha in vitro and in vivo. PloS one. 2009;4(10):e7421 10.1371/journal.pone.0007421 19823578 PMC2756622

[26] Goto T, Lee JY, Teraminami A, Kim YI, Hirai S, Uemura T, et al Activation of peroxisome proliferator-activated receptor-alpha stimulates both differentiation and fatty acid oxidation in adipocytes. Journal of lipid research. 2011;52(5):873–84. 10.1194/jlr.M011320 21324916 PMC3073464

[27] Harano Y, Yasui K, Toyama T, Nakajima T, Mitsuyoshi H, Mimani M, et al Fenofibrate, a peroxisome proliferator-activated receptor alpha agonist, reduces hepatic steatosis and lipid peroxidation in fatty liver Shionogi mice with hereditary fatty liver. Liver international: official journal of the International Association for the Study of the Liver. 2006;26(5):613–20. 10.1111/j.1478-3231.2006.01265.x . 16762007

[28] Badman MK, Pissios P, Kennedy AR, Koukos G, Flier JS, Maratos-Flier E. Hepatic fibroblast growth factor 21 is regulated by PPARalpha and is a key mediator of hepatic lipid metabolism in ketotic states. Cell metabolism. 2007;5(6):426–37. 10.1016/j.cmet.2007.05.002 . 17550778

[29] Lundasen T, Hunt MC, Nilsson LM, Sanyal S, Angelin B, Alexson SE, et al PPARalpha is a key regulator of hepatic FGF21. Biochemical and biophysical research communications. 2007;360(2):437–40. 10.1016/j.bbrc.2007.06.068 . 17601491

[30] Xu J, Lloyd DJ, Hale C, Stanislaus S, Chen M, Sivits G, et al Fibroblast growth factor 21 reverses hepatic steatosis, increases energy expenditure, and improves insulin sensitivity in diet-induced obese mice. Diabetes. 2009;58(1):250–9. 10.2337/db08-0392 18840786 PMC2606881

[31] John K, Marino JS, Sanchez ER, Hinds TD Jr. The Glucocorticoid Receptor: Cause or Cure for Obesity? American journal of physiology Endocrinology and metabolism. 2015 10.1152/ajpendo.00478.2015 . 26714851 PMC4838130

[32] Gonzalez Mdel C, Corton JC, Acero N, Munoz-Mingarro D, Quiros Y, Alvarez-Millan JJ, et al Peroxisome proliferator-activated receptoralpha agonists differentially regulate inhibitor of DNA binding expression in rodents and human cells. PPAR research. 2012;2012:483536 10.1155/2012/483536 22701468 PMC3373159

[33] Ren H, Vallanat B, Brown-Borg HM, Currie R, Corton JC. Regulation of Proteome Maintenance Gene Expression by Activators of Peroxisome Proliferator-Activated Receptor alpha. PPAR research. 2010;2010:727194 10.1155/2010/727194 21318169 PMC3026993

[34] Seo YS, Kim JH, Jo NY, Choi KM, Baik SH, Park JJ, et al PPAR agonists treatment is effective in a nonalcoholic fatty liver disease animal model by modulating fatty-acid metabolic enzymes. Journal of gastroenterology and hepatology. 2008;23(1):102–9. 10.1111/j.1440-1746.2006.04819.x . 18171348

[35] Larter CZ, Yeh MM, Van Rooyen DM, Brooling J, Ghatora K, Farrell GC. Peroxisome proliferator-activated receptor-alpha agonist, Wy 14,643, improves metabolic indices, steatosis and ballooning in diabetic mice with non-alcoholic steatohepatitis. Journal of gastroenterology and hepatology. 2012;27(2):341–50. 10.1111/j.1440-1746.2011.06939.x . 21929649

[36] Tsuchida A, Yamauchi T, Takekawa S, Hada Y, Ito Y, Maki T, et al Peroxisome proliferator-activated receptor (PPAR)alpha activation increases adiponectin receptors and reduces obesity-related inflammation in adipose tissue: comparison of activation of PPARalpha, PPARgamma, and their combination. Diabetes. 2005;54(12):3358–70. . 16306350 10.2337/diabetes.54.12.3358

[37] Zhou YT, Wang ZW, Higa M, Newgard CB, Unger RH. Reversing adipocyte differentiation: implications for treatment of obesity. Proceedings of the National Academy of Sciences of the United States of America. 1999;96(5):2391–5. 10051652 10.1073/pnas.96.5.2391 PMC26794

[38] Wang MY, Lee Y, Unger RH. Novel form of lipolysis induced by leptin. The Journal of biological chemistry. 1999;274(25):17541–4. . 10364187 10.1074/jbc.274.25.17541

[39] Lankisch TO, Moebius U, Wehmeier M, Behrens G, Manns MP, Schmidt RE, et al Gilbert's disease and atazanavir: from phenotype to UDP-glucuronosyltransferase haplotype. Hepatology. 2006;44(5):1324–32. 10.1002/hep.21361 . 17058217

[40] Cheriyath P, Gorrepati VS, Peters I, Nookala V, Murphy ME, Srouji N, et al High Total Bilirubin as a Protective Factor for Diabetes Mellitus: An Analysis of NHANES Data From 1999–2006. Journal of clinical medicine research. 2010;2(5):201–6. 10.4021/jocmr425w 21629541 PMC3104666

[41] Wu Y, Li M, Xu M, Bi Y, Li X, Chen Y, et al Low serum total bilirubin concentrations are associated with increased prevalence of metabolic syndrome in Chinese. Journal of diabetes. 2011;3(3):217–24. 10.1111/j.1753-0407.2011.00138.x . 21631904

[42] Jang BK. Elevated serum bilirubin levels are inversely associated with nonalcoholic fatty liver disease. Clin Mol Hepatol. 2012;18(4):357–9. 10.3350/cmh.2012.18.4.357 23323250 PMC3540371

[43] Kwak MS, Kim D, Chung GE, Kang SJ, Park MJ, Kim YJ, et al Serum bilirubin levels are inversely associated with nonalcoholic fatty liver disease. Clin Mol Hepatol. 2012;18(4):383–90. 10.3350/cmh.2012.18.4.383 23323254 PMC3540375

[44] Burgomaster KA, Hughes SC, Heigenhauser GJ, Bradwell SN, Gibala MJ. Six sessions of sprint interval training increases muscle oxidative potential and cycle endurance capacity in humans. Journal of applied physiology (Bethesda, Md: 1985). 2005;98(6):1985–90. 10.1152/japplphysiol.01095.2004 . 15705728

[45] Talanian JL, Galloway SD, Heigenhauser GJ, Bonen A, Spriet LL. Two weeks of high-intensity aerobic interval training increases the capacity for fat oxidation during exercise in women. Journal of applied physiology (Bethesda, Md: 1985). 2007;102(4):1439–47. 10.1152/japplphysiol.01098.2006 . 17170203

[46] Chen WC, Huang WC, Chiu CC, Chang YK, Huang CC. Whey protein improves exercise performance and biochemical profiles in trained mice. Medicine and science in sports and exercise. 2014;46(8):1517–24. 10.1249/MSS.0000000000000272 24504433 PMC4186725

[47] Arner P, Pettersson A, Mitchell PJ, Dunbar JD, Kharitonenkov A, Ryden M. FGF21 attenuates lipolysis in human adipocytes—a possible link to improved insulin sensitivity. FEBS letters. 2008;582(12):1725–30. 10.1016/j.febslet.2008.04.038 . 18460341

[48] Berglund ED, Li CY, Bina HA, Lynes SE, Michael MD, Shanafelt AB, et al Fibroblast growth factor 21 controls glycemia via regulation of hepatic glucose flux and insulin sensitivity. Endocrinology. 2009;150(9):4084–93. 10.1210/en.2009-0221 19470704 PMC2736088

[49] Chau MD, Gao J, Yang Q, Wu Z, Gromada J. Fibroblast growth factor 21 regulates energy metabolism by activating the AMPK-SIRT1-PGC-1alpha pathway. Proceedings of the National Academy of Sciences of the United States of America. 2010;107(28):12553–8. 10.1073/pnas.1006962107 20616029 PMC2906565

[50] Potthoff MJ, Inagaki T, Satapati S, Ding X, He T, Goetz R, et al FGF21 induces PGC-1alpha and regulates carbohydrate and fatty acid metabolism during the adaptive starvation response. Proceedings of the National Academy of Sciences of the United States of America. 2009;106(26):10853–8. 10.1073/pnas.0904187106 19541642 PMC2705613

